# Progressive osseous heteroplasia in a 5-year-old boy with a novel mutation in exon 2 of *GNAS*: a case presentation and literature review

**DOI:** 10.1186/s12891-023-06371-4

**Published:** 2023-03-31

**Authors:** Jing Ma, Wenxiu Mo, Jiapeng Sun, Yan Li, Tongxin Han, Huawei Mao

**Affiliations:** Department of Immunology, Ministry of Education Key Laboratory of Major Diseases in Children, Beijing Children’s Hospital, Capital Medical University, National Center for Children’s Health, No. 56 Nan Li Shi Lu, Beijing, 100045 China

**Keywords:** Progressive osseous heteropalasia, *GNAS* gene, Novel mutation, Heterotopic ossification

## Abstract

**Background:**

Progressive osseous heteroplasia (POH) is a rare genetic condition that causes progressive ossification. This usually results from an inactivating mutation of the paternal *GNAS* gene. Herein, we report a case of POH caused by a novel mutation in exon 2 of the *GNAS* gene.

**Case presentation:**

A 5-year-old Chinese boy was referred to our hospital for a growing mass in his right foot. Although laboratory findings were normal, radiographic imaging revealed severe ossification in his right foot and smaller areas of intramuscular ossification in his arms and legs. A de novo mutation (c.175C > T, p.Q59X) in exon 2 of the *GNAS* gene was identified, prompting a diagnosis of POH. We conducted a systematic literature review to better understand this rare disease.

**Conclusion:**

We have discovered that a de novo nonsense mutation in exon 2 of *GNAS* can lead to POH. Our literature review revealed that ankylosis of the extremities is the primary clinical outcome in patients with POH. Unlike other conditions such as fibrodysplasia ossificans progressiva (FOP), patients with POH do not experience respiratory failure. However, much remains to be learned about the relationship between the type of GNAS gene mutation and the resulting POH symptoms. Further research is needed to understand this complex and rare disease. This case adds to our current understanding of POH and will contribute to future studies and treatments.

**Supplementary Information:**

The online version contains supplementary material available at 10.1186/s12891-023-06371-4.

## Background

Progressive osseous heteroplasia (POH) is an extremely rare genetic disorder characterised by progressive formation of heterotopic ossification in deep connective tissues and skeletal muscles [1]. POH’s severity can range from subcutaneous lesions to complete ankylosis of the involved joints [2, 3]. It is primarily caused by inactivating mutations of the paternal *GNAS* gene. The *GNAS* gene is located on chromosome 20q13.3 and encodes the alpha subunit of the stimulatory G protein (Gsα) and other imprinted transcripts [2, 4, 5]. Heterozygous inactivating mutations of *GNAS* are also responsible for Albright’s hereditary osteodystrophy (AHO) [2], which includes a constellation of symptoms: brachydactyly, stocky build, short stature, round face ectopic ossification, and intellectual disability [6, 7]. In POH and AHO, heterotopic ossification primarily occurs via intramembranous ossification [5]. Overlapping syndromes, such as POH/PHP1A and POH/PPHP, have also been reported [8–11]. POH and AHO are considered as part of the same spectrum of GNAS-related ossification diseases. However, compared to AHO, POH is considered a more severe form of the disease as it is associated with greater ossification.

Since its initial description in 1994 [12], apart from one large cohort [13], only a few individual cases and case series have been published worldwide. This has limited our ability to better understand this rare disease. Herein, we present a case of POH with a novel mutation in exon 2 of *GNAS*.

## Case presentation

On April 02, 2021, a 5-year-old Chinese boy visited our hospital with complains of a progressively growing mass under the skin of his right foot for 1 year. The boy was born at full-term after an uncomplicated pregnancy with a birth weight of 2.8 kg. At 4 years of age, his parents noticed a hard mass under his right foot, with no signs of redness or swelling over the skin. The family visited a local hospital, where radiographs revealed ossification of the right foot. Further investigations were recommended, however, the patient’s parents refused and requested only observation at the time. Over the following year, the mass grew, however, it was manageable during mechanical loading. The boy was healthy prior to this.

Physical examination at our hospital revealed that the boy was 102.5 cm tall (-2 SD) and weighed 14.5 kg (-2 SD). He was a slim, right-handed boy with Tanner I pubertal development and no signs of mental retardation or developmental delay. A comprehensive subcutaneous mass was palpable in sole of the right foot, which extended to the Achilles tendon and caused minimal limitations in ankle movement. The mass could not be precisely localised. The remainder of the physical examination results were normal.

**Fig. 1:**
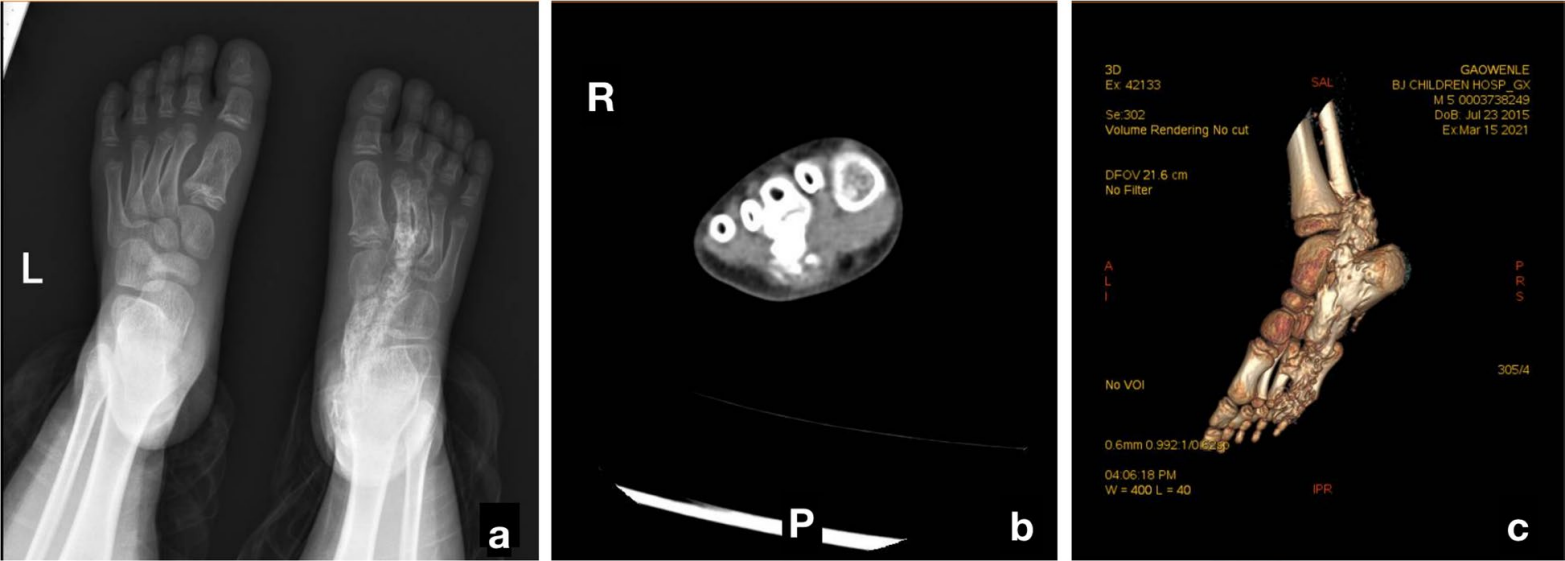
The radiographic image of the foot. AP right foot radiograph shows massive ossification centered along the third metatarsal from the toes to the hind foot. Some of the ossified areas have fused together (**a**). Axial CT of the patient shows ossification on the plantar aspect of the right foot extending between the second and third metatarsals with incorporation of the ossification in the third metatarsal (**b**). The tridimensional CT scan of the lower extremities reveled the range of the right foot calcifications (**c**)

**Fig. 2:**
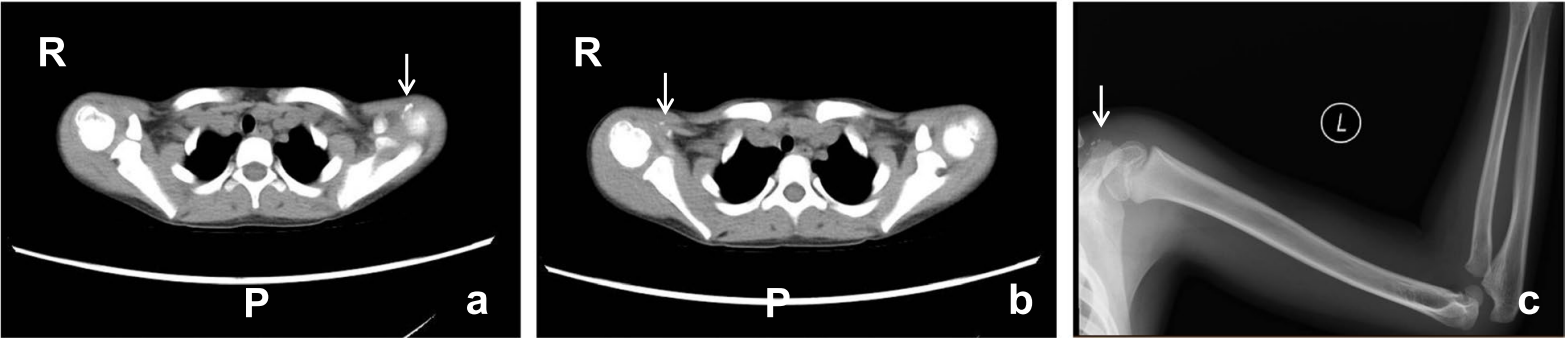
The radiographic image of the shoulders. The radiographic image of the shoulder revealed tiny radiodensities (arrows) seen in the skeletal muscle of bilateral proximal humerus (**a, b**) and left acromial area (**c**)

**Fig. 3:**
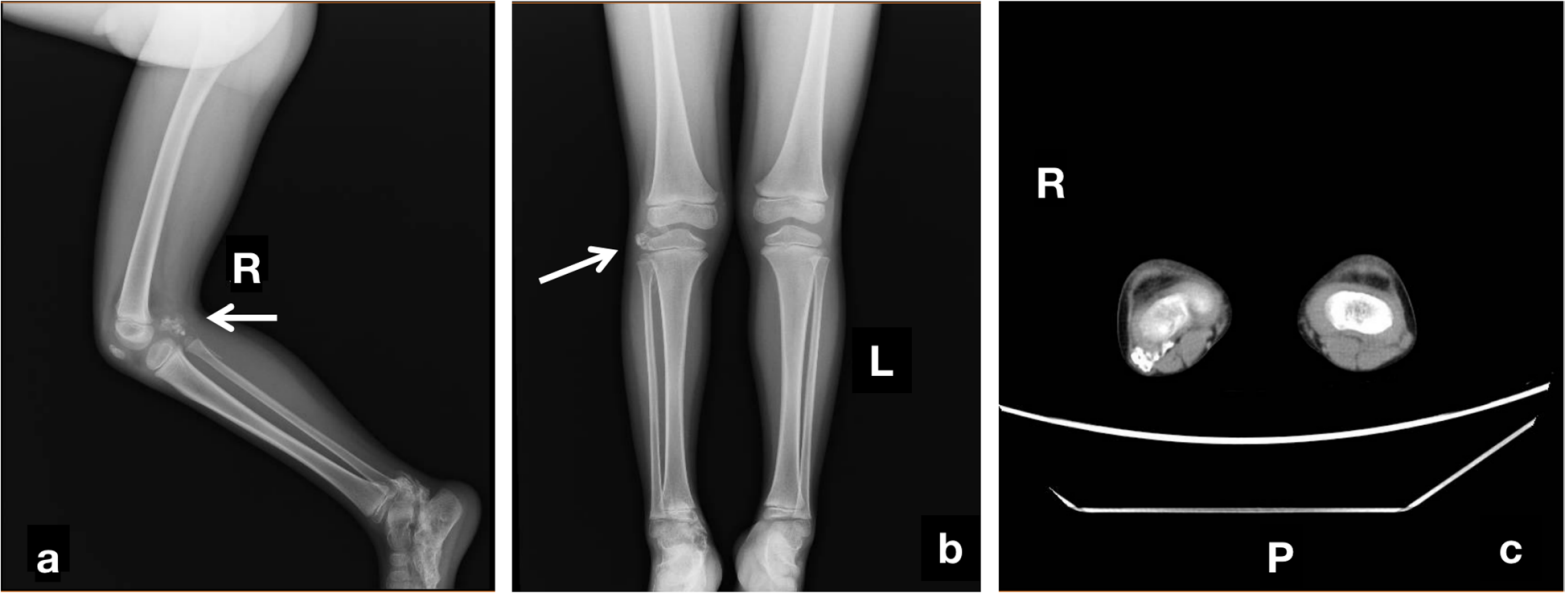
Radiographs of the right keen. Lateral and anteroposterior radiograph of the legs shows ossification (arrows) in the soft connective tissues from the dermis down through the muscle to the proximal tibi (**a, b**). Axial CT shows ossification has fused with the tibia (**c**)

The serum levels of calcium, phosphorus, and alkaline phosphates were found to be normal. An anteroposterior radiograph of the right foot showed long sheets of opacification across the plantar aspect of the foot and heel, some opacified sheets were continuous with metatarsals and tarsal bones (Fig. 1). Axial computed tomography (CT) images revealed ossification in the right foot between the second and third metatarsals, which was continuous with the foot bones. Three-dimensional CT revealed progression of the ectopic ossification (Fig. 1). X-ray screening was performed to identify other sites of heterotopic ossification, small areas of intramuscular ossification were observed at the superior margin of bilateral proximal humerus (Fig. 2). Additionally, a limited radio-dense lesion was noted in the right popliteal fossa (Fig. 3).

Soft-tissue ossification-causing rheumatic diseases, such asjuvenile dermatomyositis, systemic lupus erythematosus, and systemic sclerosis, were ruled out as the patient showed no systemic clinical features and tests for autoantibodies were negative. There was a high suspicion of fibrodysplasia ossificans progressiva (FOP) due to the massive heterotopic ossification. FOP was ruled out because the patient showed no signs of inflammatory soft tissue swelling or malformed great toes. The large heterogeneously ossified mass, progressive nature of the lesions, and normal laboratory findings prompted a clinical diagnosis of POH. AHO was unlikely, as the patient did not exhibit any other characteristic anomalies of AHO despite the short stature.

Whole-exome sequencing was performed to confirm the diagnosis. A de novo heterozygous inactivating mutation (c.175C > T, p. Q59X) was identified in exon 2 of the *GNAS* gene. This is the first reported case of such a mutation leading to POH. Physical therapy was recommended to preserve movement. The ossified lesion continued to grow slowly over the following year.

**Fig. 4:**
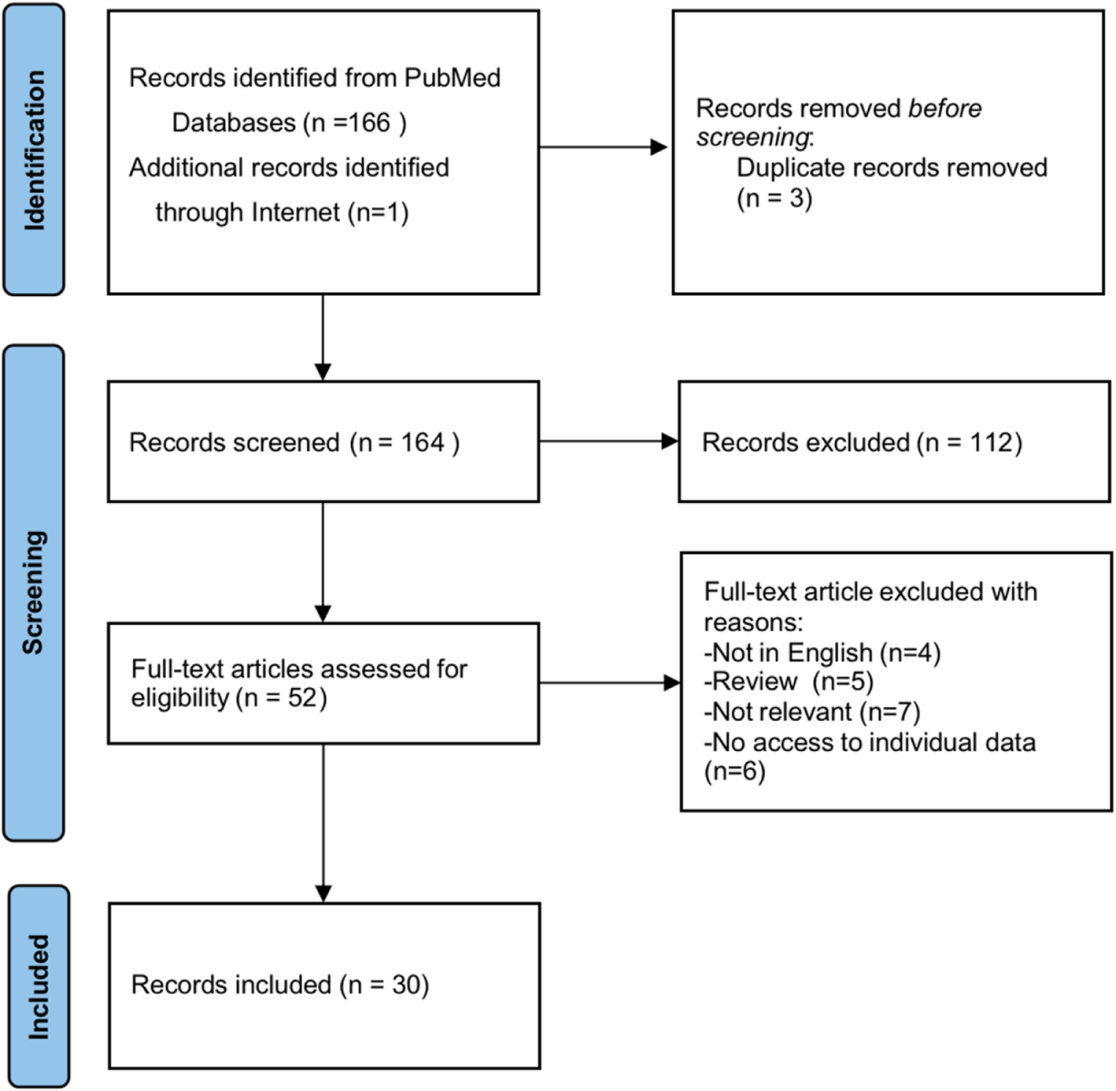
Flow of selection of articles for the systematic literature review

We performed a comprehensive literature search using the search string: (Progressive Osseous Heteroplasia) OR ((Heterotopic Ossification) AND GNAS)) AND ("1999/12/31"[Date—Publication]: "2022/01/01"[Date—Publication]). A total of 166 articles were retrieved from PubMed, one additional article was identified through an online search engine. After eliminating duplicates, 164 studies were included. Subsequently, 112 articles were deemed irrelevant based on the title and abstract and were excluded. Thereafter, 22 articles were excluded for various reasons (Fig. 4). Finally, 30 articles, which included 40 patients (22 female), were analyzed, the clinical and genetic features are summarized in (Additional file 1: Table 1) [3, 8, 10, 11, 14–39].

## Discussion and conclusion

POH is an extremely rare genetic disorder that results in progressive heterotopic ossification. We identified a de novo mutation (c.175C > T, p.Q59X) in the *GNAS* gene in a young Chinese boy with typical features of POH. Although it was not determined which parental allele carried the mutation, the lack of AHO features and the presence of hormone resistance suggested that it was carried on the paternal allele.

A comprehensive literature review was conducted to gain a deeper understanding of the disease. POH commonly presents in infancy and childhood (average onset age 2.34 years), with only one patient having adult-onset ossification. Patients with POH are often diagnosed late owing to the rarity of the disease and its gradual onset. The age at onset in our patients may have been earlier than 4 years, as the ossification sites are difficult to detect. POH can be categorised as widespread heterotopic ossification with no functional impairment or progressive heterotopic ossification resulting in complete ankylosis. Our patient seemed to belong to the first category, as no severe ankylosis was found. In most patients with POH, ossification was predominantly seen in the extremities (83%, 34/41) and lower back (34%, 14/41), with only four patients (4/41, 10%) having ear and mandible involvement. Unilateral involvement was encountered in 46% (19/41) of patients and bilateral asymmetrical involvement in 64% (22/41) of the patient. Unlike FOP, none of the patient with POH suffered from dyspnoea due to respiratory muscle involvement. The predominant clinical outcome in patients with POH is ankylosis of the limbs, the trunk is not involved. One third of the patients with POH (12/41) had more than one clinical feature of AHO with or without hormonal resistance, supporting the notion that POH represents one end of the spectrum of *GNAS*-related diseases.

**Fig. 5:**
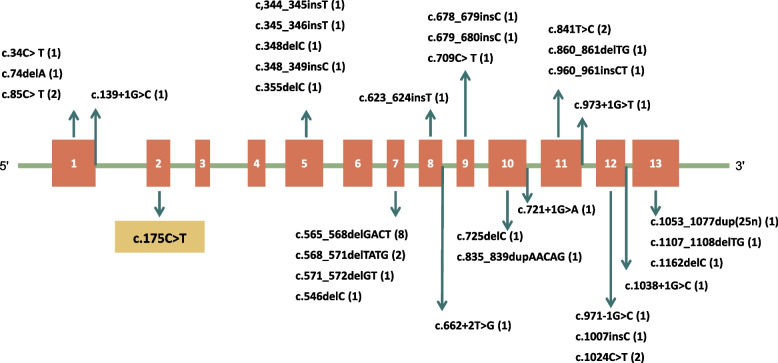
GNAS mutations in patients with POH. This schematic represents the GNAS gene: Exons are indicated by boxes with number. Intronic sequences are indicated by solid lines between exons. The box with yellow shade indicates the mutation identified in this case report. Additional file 1: Table 1 did not show all mutations for detail clinical features were lacking. The number of times each variant are showed in the parentheses

*GNAS* gene mutations (Fig. 5) were found in only 66% (27/41) of patient with POH, with a four-base pair deletion of c.565–568 being present in 17.10% (7/41) of the patients. This deletion “hotspot” has been reported in patients with other *GNAS*-related diseases, such AHO, PHP1A, and OC [13]. The same *GNAS* mutation could cause both types of ossifications, with no specific distribution pattern. A unique case of POH was reported in two monochorionic twins with the same mutation and genetic background, however, the clinical presentations differed [25]. Two patients with POH and without *GNAS* mutations had a family history of heterotopic ossification and/or hormone resistance [15, 16], indicating that POH is influenced by various factors in addition to genetics. Further research is needed to explore the role of unknown epigenetic modifications and environmental factors in ectopic ossification.

Currently, no medication can effectively reverse or prevent ossification associated with POH. Recurrence of lesions in POH following resection is common, and the recurrent lesions tend to be larger in size [35, 40, 41]. We recommended meticulous skin care and physical therapy [1]. POH management is supportive, with a focus on educating patients and improving their quality of life. Recently, Qian et al. [42] discovered a self-amplifying, self-propagating loop of Yes-associated protein (YAP) and Sonic hedgehog (SHH) that plays a key molecular role in the development of ossification in POH. These results suggest that the inhibition of YAP or SHH could prevent, reduce, and shrink POH lesion, while maintaining normal bone homeostasis. Although a few patients have been followed-up, the long-term prognosis of POH remains uncertain. The disease stabilised or progressed at a slower rate in the patients who were followed-up [4]. In one case reported by Ezzat El Sobky et al., a patient with POH succumbed to death due to a severe secondary infection of the skin ulcers overlying the subcutaneous ossification [39]. Further research of the disease is required. Given the progressive nature of POH, it is essential to closely monitor affected young individuals, as their phenotype may become more complex over time.

In conclusion, POH is an extremely rare condition characterised by progressive ossification of the skin, subcutaneous tissue, and connective tissue, leading to immobility in severe cases. POH is caused by a genetic mutation, specifically an inactivating mutation of the *GNAS* gene located on the paternal allele. This report describes the case of a boy with typical POH symptoms and radiographic findings, in whom a de novo mutation was detected in exon 2 (c.175C > T, p.Q59X) of the *GNAS* gene. A systematic literature review revealed that limb ankylosis is a common outcome in patient with POH. Unlike in FOP, there have been no reports of respiratory failure in patients with POH due to ossification of respiratory muscles. Our literature review also revealed no clear relationship between the genetic mutations and the clinical manifestations. Through this report we aimed to increase awareness and understanding of this rare disorder among healthcare professionals, including physicians, paediatricians, and orthopaedic surgeons. We recommend avoiding unnecessary treatment and surgeries and providing proper counselling for patients and families.

## Supplementary Information

**Additional file 1: Table 1.** The clinical and genetic features of POH.

## Data Availability

Data sharing does not apply to this article, as no datasets were generated or analyzed during the current study.

## Abbreviations

POH: Progressive osseous heteroplasia
FOP: Fibrodysplasia ossificans progressive
HO: Heterotopic ossification
Gsα: The alpha subunit of stimulatory G protein
PHP1A: Pseudohypoparathyroidism type 1A
PPHP: Pseudopseudohypoparathyroidism
AHO: Albright’s hereditary osteodystrophy
PTH: Parathyroid hormone
TSH: Thyroid-stimulating hormone
OC: Osteoma cutis
AP: Anteroposterior
CT: Computed tomography
ACMG: American College of Medical Genetics and Genomics
NESP55: Neuroendocrine Secretory Protein 55
XLαs: Extralarge Gα
cAMP: Cyclic adenosine monophosphate
YAP: Yes-associated protein
SHH: Sonic hedgehog
