# Supplementary material for: Progressive osseous heteroplasia in a 5-year-old boy with a novel mutation in exon 2 of GNAS: a case presentation and literature review
Source: BMC Musculoskelet Disord. 2023 Mar 31;24:247. doi: 10.1186/s12891-023-06371-4 (PMC10064707; doi:10.1186/s12891-023-06371-4)
Supplement: Supplementary file 1 — Additional file 1: Table 1. The clinical and genetic features of POH. [file 12891_2023_6371_MOESM1_ESM.docx]

Table 1. The clinical and genetic features of POH

| **PT** | **Author** | **G** | **AO** | **AD** | **Superficial**  **ossification** | **Progressive**  **ossification** | **Ankylosis** | **AHO**  **features** | **Mutation** | **Inheritance/**  **mutated allele** |
| --- | --- | --- | --- | --- | --- | --- | --- | --- | --- | --- |
| **2009** | Schimmel RJ [35] | F | 11d | 8y | L upper limb | L upper limb | L upper limb | HO | c.1107–1108delTG  (NA) | *De novo* |
| **2010** | Goto M  [23] | M | 21m | 6y | R heel | R heel |  | HO SS | c.1024C>T  (p.R342X) | *De novo*  paternal |
| **2015** | Lin MH  [27] | M | 18m | 3y | L lower limb | L lower limb |  | HO  TSH↑ PTH↑ | c.1024C>T  (p.R342X) |  |
| **2018** | Pereda A [29] | F | 4y | 32y | R upper limb | R upper limb | R upper limb | HO | c.973+1G>T*  (NA) | *De novo* |
| **2003** | Faust RA  [22] | F | 5m | 5m | L mandible | L mandible | dysphagia | HO | c.860_861delTG  (NA) |  |
| **2006** | Hou JW  [24] | F | birth | 5.5y | chest, abdomen, lower limbs |  |  | HO | c.841T>C  (p.W281R) | *De novo* |
| **2004** | Chan I  [18] | F | 9m | 9y | trunk lower limbs | R calf |  | HO | c.841T>C  p. W281R | *De novo* |
| **2022** | Chang G  [19] | M | birth | 0.8y | L thigh shanks, back, ear, inframandibular region,  L wrist |  |  | HO | c.721+1G>A  (NA) | *De novo* |
| **2021** | Han SR  [10] | F | NA | 6.5y | L foot | L foot |  | HO | c.709C> T  p.Q237X | *De novo* |
| **2010** | Lebrun M  [14] | M | 3m | 5y | widespread dorsal | sacrum and coastal drill |  | HO | c.623_624insT  (NA) | *De novo* /  paternal |
| **2010** | Lebrun M  [14] | M | 3y | 9y | hands  knees | quadriceps muscle |  | HO | c.571_572delGT  (NA) | *De novo* /  paternal |
| **2016** | Mariani M  [3] | F | 7y | 7y | parasternal region  R popliteal  L ankle |  |  | OC RF | c.568_571delTATG  (NA) | *De novo* |
| **2008** | Kumagai K [26] | M | 4y | 9y | R side back  R ear helix | R side back |  | HO | c.565_568delGACT  p.D189Mfs*14 | *De novo* |
| **2021** | Justicia-Grande AJ [25] | F | 10w | 4y |  | lower limbs  back | lower limbs | HO | c.565_568delGACT  p.D189Mfs*14 | *De novo* |
|  |  |  |  |  |  |  |  |  |  |  |
| **2010** | Lebrun M  [14] | M | 3m | 3m | multiple (largest 8cm) | invading deeper tissues |  | HO RF BR | c.565_568delGACT  p.D189Mfs*14 | *De novo* /  paternal |
| **2010** | Lebrun M  [14] | F | 4y | 7y | multiple (largest 14cm) | invading deeper tissues |  | HO | c.565_568delGACT  p.D189Mfs*14 | *De novo* /  paternal |
| **2021** | Schepis C [32] | F | 7m | 7m | limbs and trunk |  |  | HO | c.565_568delGACT  p.D189Mfs*14 | *De novo* |
| **2021** | Ozaki K  [11] | M | 5y | 13y | R hand  L foot |  |  | HO SGA  TSH↑ PTH↑ | c.565_568delGACT  p.D189Mfs*14 | *De novo* |
| **2014** | Schrander DE [34] | F |  | 7y | L foot  Lumber spine  L scapulae |  |  |  | c.565_568delGACT  p.D189Mfs*14 |  |
| **2007** | Gelfand IM [8] | F | 1m | 4m | R lower limb | R lower limb | knee | BR HO RF TSH↑  PTH↑ | c.546delC  (NA) | *De novo* |
| **2010** | Lebrun M  [14] | F | birth | birth |  | severe, extensive | hemi-hypotrophy | HO | c.345_346insT  (NA) | *De novo* /  paternal |
| **2010** | Lebrun M  [14] | F | 1y | 11y | superficial and local |  |  | HO BR | c.139+1G>C  (NA) | *De novo* /  paternal |
| **2010** | Lebrun M  [14] | M | NA | 11y | knees, hands, feet | knees, hands, feet |  | HO | c.85C> T  p.Q29X | De novo/  paternal |
| **2010** | Lebrun M  [14] | F | 2y | 52y | knees, hands, wrist, buttocks, abdomen, feet | knees, hands, wrist, buttocks, abdomen, feet |  | HO | c.85C> T  p.Q29X |  |
| **2018** | Zhang SD [38] | M | birth | 1y | R wrist  abdomen  back  R knee  L ear | R wrist |  | HO | c.74delA  p.K25Kfs*33 | *De novo* |
| **2000** | Eddy MC  [21] | F | 3m | 10y | R ear,  R lower limb |  |  | HO SS BR | c.34C>T  p.Q12X | De novo |
| **2000** | Eddy MC  [21] | F | 6m | 12y | abdomen, and extremities | R lower limb | R ankle  R foot | HO BR RF SB MR | None |  |
| **2002** | Aynaci O  [16] | F | 40d | 5y | Upper limbs | Upper limbs | Upper limbs | HO | None |  |
| **2020** | Sahu K  [30] | M | 2m | 3y | R lower legs | R thigh |  | HO | None |  |
| **2021** | Justicia-Grande AJ[25] | F | 10m | 4y | tights | R leg  back  iliac fossae | R leg | HO | None |  |
| **2020** | Miles O [28] | M |  | 24y | R hand | first MCP |  | HO | None |  |
| **2021** | Zhang K  [37] | F | 3m | 9y | R face  R neck | R face  R neck  R knee  L shoulder  R iliac spine | L shoulder  head  neck | HO | None |  |
| **2009** | Santiago F [31] | M | <1y | 50y | L forearm  L lower limb | L forearm  L lower limb | L ankle | HO | None |  |
| **2015** | Lin MH  [27] | M | 6m | 4y | R gluteal  R forearm | R thigh  R gluteal  R forearm |  | HO SB RF BR | None |  |
| **2015** | Lin MH  [27] | M | 4y | 8y | torso  knees | torso  shoulders | knees | HO | None |  |
| **2019** | Arrigoni P [15] | F | <1y | 16y | feet  back | R foot  L gluteus fascia |  | HO BR SS | None |  |
| **2016** | Birjandinejad A [17] | M | 1.5y | 22y | R upper limb | R upper limb | R upper limb | HO | None |  |
| **2011** | Singh GK  [36] | M | 3m | 10y | L upper limb | L upper limb | L upper limb | HO | None |  |
| **2007** | Seror R  [35] | F | 36y | 52y | hands, elbows, lower limbs | four limbs, paravertebral |  | HO | None |  |
| **2010** | El Sobky E [39] | M | 2y | 5y | four limbs | four limbs | four limbs | HO | NA |  |
| **2015** | Demir MK [20] | M |  | 19y | scapula  dorsal  lumbarspine  gluteal | scapula  dorsal  lumbarspine  gluteal | lumber  scoliosis | HO | None |  |
| **PT, published time; AO, age of onset; AD, age of diagnose; SS, short stature; BR, brachydactyly; HO, heterotopic ossification; RF, round face; SB stocky build; MR, mental retardation; NA, not available. All the variants involve the same transcript (NM_000516) except one (c.973+1G>T, NM_001077488) marked by asterisk (*).** | | | | | | | | | | |
